# Renoprotective effects of levosimendan on acute kidney injury following cardiac arrest via anti‐inflammation, anti‐apoptosis, and ERK activation

**DOI:** 10.1002/2211-5463.13227

**Published:** 2021-07-12

**Authors:** Lei Tian, Shiwei Wang, Li Zhao, Xiaoye Lu, Changqing Zhu, Hao Gong, Weiqiang Yang

**Affiliations:** ^1^ Department of Emergency School of Medicine Renji Hospital Shanghai Jiao Tong University China

**Keywords:** acute kidney injury, cardiac arrest, cardiopulmonary resuscitation, ERK, levosimendan

## Abstract

ATP‐sensitive potassium channels (KATPs) have protective effects in ischemia–reperfusion‐induced injuries and can be activated by levosimendan. This study investigated the effects of levosimendan on renal injury, inflammation, apoptosis, and survival in a rat model of acute kidney injury (AKI) following cardiac arrest (CA) and cardiopulmonary resuscitation (CPR). Rats underwent a 5‐min asphyxia‐based CA and resuscitation. The rats were treated with levosimendan after successful resuscitation. Renal functions, histological changes, inflammatory responses, and apoptosis were examined. NRK‐52E cells treated by hypoxia/reoxygenation (H/R) were used to establish an *in vitro* CA‐CPR model. Rats in the CA‐induced AKI group had a low survival rate and increased levels of creatinine, blood urea nitrogen, and proinflammatory cytokines, as well as increased tubular injury. These results were significantly reversed after treatment with levosimendan. Levosimendan downregulated the expression of the apoptosis‐related proteins Bax, cleaved caspase‐3, and cleaved caspase‐9, as well as upregulated Bcl‐2 and p‐ERK expression *in vivo* and *in vitro*. Thus, our data suggest that levosimendan reduces mortality and AKI following CA and CPR via suppression of inflammation and apoptosis, and activation of ERK signaling.

## Graphical abstract

Acute kidney injury (AKI) is an important morbidity of cardiac arrest (CA). Levosimendan, a nonselective ATP‐sensitive potassium channel (KATP) activator, could alleviate AKI following CA and cardiopulmonary resuscitation (CPR) via suppression of inflammation and apoptosis and activation of ERK signaling.

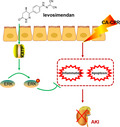

**Abbreviations**
AKI: acute kidney injury
CA: cardiac arrest
CPR: cardiopulmonary resuscitation
IL‐1β: interleukin 1β
IL‐6: interleukin 6
IRI: ischemia–reperfusion injury
KATPs: ATP‐sensitive potassium channels
p‐ERK: phospho‐extracellular signal‐regulated kinase
p‐JNK: phospho‐c‐Jun N‐terminal kinase
ROSC: return of spontaneous circulation
TNF‐α: tumor necrosis factor‐α

Cardiac arrest (CA) remains a major worldwide public health burden in terms of high mortality rates and lethal sequelae [1, 2]. Cardiopulmonary resuscitation (CPR) is the most urgent and critical step in the treatment of patients with CA [3]. The development of CPR technology has significantly improved the success rate of the return of spontaneous circulation (ROSC) in patients with sudden CA. ROSC occurs in a maximum of approximately 10% of patients with CA; furthermore, even CA survivors with ROSC often die in the emergency department within 24 h, and the survival‐to‐discharge rate at the hospital is only approximately 10% [4, 5].

Cardiac arrest and subsequent CPR are complex processes of ischemia–reperfusion (I/R) [6]. One of the most vulnerable organs during CA and subsequent CPR is the kidney. Post‐resuscitation acute kidney injury (AKI) is an important morbidity of post‐resuscitation syndrome, which is closely related to ischemia–reperfusion [7]. AKI may occur in 12%–81% of adult CA patients and is associated with poor prognosis [8].

ATP‐sensitive potassium channels (KATPs) have protective effects in ischemia–reperfusion‐induced injury, are abundantly expressed in the kidney, and are activated by levosimendan [9]. Grossini *et al*. reported that levosimendan attenuated ischemia–reperfusion‐induced renal injuries by ameliorating oxidative stress, apoptosis, and inflammation in pigs [10]. In our previous study [11], levosimendan was shown to be protective against AKI following CA and CPR. However, the exact mechanisms are far from clear.

In this study, the effects of levosimendan on AKI, inflammation, and apoptosis following CA and CPR were examined. The aim of this study was to determine whether levosimendan prevents AKI via anti‐inflammatory and anti‐apoptotic activities.

## Materials and methods

### Animal and drugs

All animal experiments were performed using a protocol approved by Renji Hospital, School of Medicine, Shanghai Jiao Tong University. Sprague‐Dawley male rats weighing 450–500 g at the age of 15–16 weeks were obtained from Shanghai SLAC Laboratory Animal. Rats had free access to food and water.

Pentobarbital sodium was purchased from Sigma Chemical (St. Louis, MO, USA). Levosimendan was purchased from Qilu Pharmaceutical Co Ltd (Jinan, China).

### Experimental procedures

We established the 5‐min asphyxia CA‐CPR model based on previous method with a slight modification [12]. Each rat was anesthetized with pentobarbital sodium solution (45 mg·kg^−1^). When the rat showed no signs of sensation, a 14‐G catheter was inserted into the trachea with oral assistance by a laryngoscope. To maintain anesthesia and reduce suffering, 10 mg·kg^−1^ pentobarbital sodium was given by intraperitoneal injection every hour or when necessary. At the end of the 5‐min asphyxia procedure, CPR was initiated by unclamping the tracheal tube and reconnecting the ventilator to 100% oxygen for inhalation, injecting epinephrine (0.02 mg·kg^−1^) and sodium bicarbonate (1 mEq·kg^−1^), and applying external chest compressions (200 compressions·min^−1^). Successful ROSC was defined as an initial return of sinus electrocardiogram rhythm and blood pressure exceeding 60 mm Hg that lasted for at least 5 min. The clamp on the tracheal tube was released immediately after 5 min of asphyxia.

After achieving ROSC, the animals were randomized into the levosimendan treatment group (levo, *n* = 20) and the asphyxia group (Asp, *n* = 20). In addition, the animals in the sham group (*n* = 10) underwent the same operation but without inducing CA. The rats in the levo group received 12 μg·kg^−1^ levosimendan intravenously after 0.5 min of ROSC and then a continuous intravenous infusion of levosimendan (0.3 μg·kg^−1^·min^−1^). The mean arterial pressure (MAP) of the rats in the sham, Asp, and levo groups was continuously recorded for 24 h. Twenty‐four hours after ROSC was achieved, the rats were sacrificed. Serum was separated by centrifugation at 600 g for 15 min at 4 °C and stored at –80 °C until use. Renal samples were immediately fixed in 4% formaldehyde and used for histological analysis or rinsed in saline buffer and stored at –80 °C until use. The animal experiment was approved by Renji Hospital Committee and complied with ethical regulations.

### Survival rate assessment

In each group, the survival rate was assessed over a period of 48 h after sham operation or CA‐CPR. Following CA‐CPR, the rats were carefully observed every 6 h for 48 h. The time of mortality was recorded.

### Assessment of renal function and histologic injury

Serum samples were collected and used for detecting serum creatinine (Scr) and blood urea nitrogen (Bun) by an autoanalyzer (Roche Diagnostics GmbH, Mannheim, Germany). Kidney tissues were fixed with 4% paraformaldehyde and embedded in paraffin. Kidney sections were stained with hematoxylin and eosin (HE) and then assessed under a microscope. Tubular injury score was quantified by the percentage of damaged tubules as previously described [13]: grade 0, no damage; grade 1, <25%; grade 2, 25%–49%; grade 4, 50%–74%; grade 4, ≧75%. Tubular injury score was evaluated in 10 randomly selected, nonoverlapping fields at 100× magnification in each section.

### Immunohistochemistry

Paraffin‐embedded kidney was cut into section of 5‐μm thickness, and a standard protocol, using xylene and graded ethanol, was used to deparaffinize the tissue. After washing with PBS, sections were microwaved in Tris–EDTA buffer (Maxim Biotechnologies, Shanghai, China) and cool to room temperature. They were then treated with blocking buffer containing 5% normal donkey serum (Jackson ImmunoResearch Laboratories, West Grove, PA, USA) for 60 min at room temperature. The sections were incubated with an anti‐CD68 (1 : 100, Proteintech, Wuhan, China) or anticleaved caspase‐3 (1 : 100, Proteintech) primary antibody at 4 °C overnight and then with a horseradish peroxidase‐labeled secondary antibody (Beyotime, Shanghai, China). Quantitative analysis of positive staining area was evaluated randomly in 10 high‐power fields.

### Cytokine analysis of kidney homogenization

Kidney homogenization was performed as described previously [14]. The levels of interleukin 1β (IL‐1β), interleukin 6 (IL‐6), and tumor necrosis factor‐α (TNF‐α) in kidney homogenization were determined by ELISA, according to the manufacturer’s instructions (Proteintech).

### Cell culture and treatment

NRK‐52E cells were cultured in RPMI‐1640 medium (GIBCO, New York, NY, USA) containing 10% FBS (GIBCO) in a humidified incubator at 37 °C with 5% CO_2_. Post‐resuscitation AKI is closely related to I/R [6]. I/R injury in the cells was simulated based on an *in vitro* hypoxia/reoxygenation (H/R) model. NER‐52E cells used in our experiments were divided into the groups as follows: control, H/R, and levosimendan (levo). Normal NRK‐52E cells were used as the control group and were treated as previously described. For the H/R group, NRK‐52E cells were exposed to hypoxia containing 5% CO_2_, 1% O_2_, and 94% N_2_ for 12 h, followed by 24 h of reoxygenation with 5% CO_2_, 21% O_2_, and 74% N_2_. For the levo group, NRK‐52E cells received the same treatment as the H/R group, except that they were precultured in 5% CO_2_ with 2% levosimendan for 1 h before culture in an incubator.

### Immunoblotting analysis

For immunoblot analysis, renal tissues and NEK‐52E cells were lysed using the RIPA buffer containing protease inhibitor cocktail (Santa Cruz, Carlsbad, CA, USA) and quantified with BCA kit (Thermo Fisher, Waltham, MA, USA). Protein extracts were separated in sodium dodecyl sulfate/polyacrylamide gel electrophoresis, transferred to polyvinylidene difluoride membranes, and incubated with the primary antibodies. The antibodies used were as follows: anti‐GAPDH (1 : 1000, Proteintech), anticleaved caspase‐3(1 : 1000, Proteintech), anticleaved caspase‐9 (1 : 1000, Proteintech), anti‐Bcl‐2 (1 : 1000, Proteintech), anti‐Bax (1 : 1000, Proteintech), anti‐phospho‐p38 (1 : 1000, Proteintech), anti‐phospho‐c‐Jun N‐terminal kinase (p‐JNK; 1 : 1000, Proteintech), and anti‐phospho‐extracellular signal‐regulated kinase (p‐ERK; 1 : 1000, Proteintech).

### Statistics

Quantitative data shown in this study are representative of at least three separate experiments. Quantitative data were expressed as means ± standard error of the mean (SEM). Student's *t*‐test was used to assess the difference between two groups. One‐way ANOVA was used to analyze the difference among three or more groups, followed by *post hoc* analysis. Statistical analysis was performed using graphpad software (version 6.0, GraphPad Software, La Jolla, CA, USA). Significant values of *P* < 0.05 were considered.

## Results

### Hemodynamic parameters at 24 h post‐resuscitation

At 24 h post‐resuscitation, MAP values in Asp group and levo group were significantly lower than those of the sham group (*P* < 0.05). However, there was no significant difference in MAP between the Asp group and levo group (Table 1). Thus, we observed no effect of levosimendan on peripheral blood pressure in rat model of CA.

**Table 1:** Hemodynamic parameters at 24 h post‐resuscitation. MAP, mean aortic pressure; values are presented as mean ± SD

Group	sham	Asp	levo
Body weight, g	505 ± 12	507 ± 14	510 ± 20
Heart rate, bpm	329 ± 8	324 ± 10	326 ± 10
MAP, mm Hg	128.6 ± 3.0	84.6 ± 2.1*	87.6 ± 1.2*

* *P* < 0.05, vs. sham. Student's *t*‐test was used to assess the difference between two groups. *P* value < 0.05 was considered statistically significant.

### Survival

**Fig. 1:**
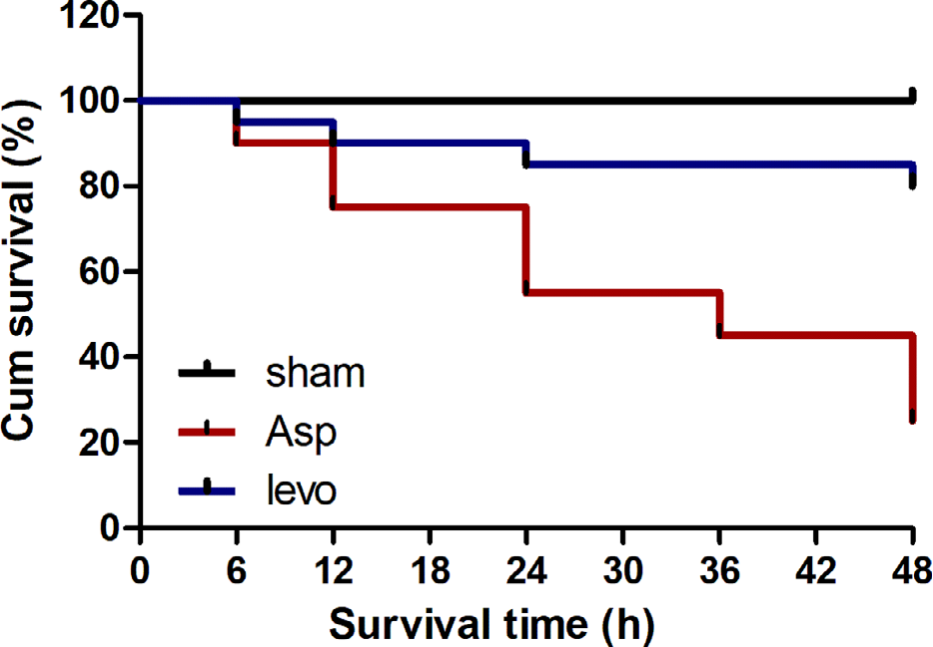
Levosimendan increases survival after CA and CPR. CA in rats was induced by 5 min asphyxia. Survival of rats was monitored for 48 h after CA (*n* = 20 per group). Asp, CA‐CPR group; levo, levosimendan treatment group.

We assessed mortality by monitoring survival for 48 h. After CA‐CPR, the survival of rats was 90% at 6 h, 75% at 12 h, 55% at 24 h, 45% at 36 h, and 25% at 48 h. The survival of rats in levo group was 95% at 6 h, 90% at 12 h, 85% at 24 h, 85% at 36 h, and 80% at 48 h (Fig. 1). Therefore, levosimendan improved survival of rats after CA‐CPR.

### Levosimendan treatment ameliorates CA‐induced renal dysfunction and renal injury

**Fig. 2:**
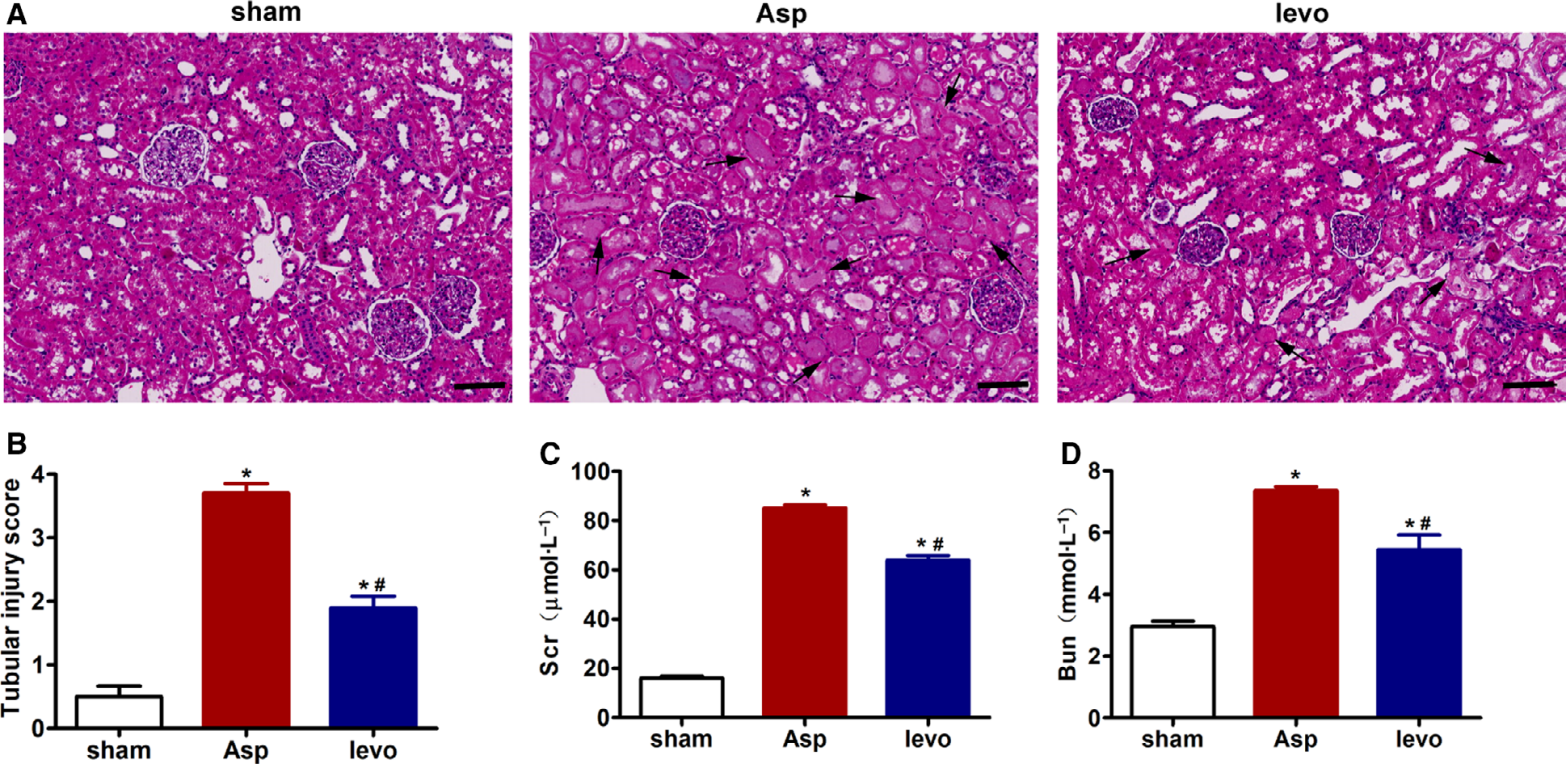
Levosimendan treatment ameliorates CA‐induced renal dysfunction and renal injury. (A) Representative micrographs of H&E staining of kidney sections (scale bar 100 μm) and (B) quantitative data of tubular injury score. Arrows indicate positive staining. (C) Serum creatinine (Scr) and (D) blood urea nitrogen (Bun) at 24 h after CA‐CPR. Data represent mean±SEM, *n* = 3–6. Asp, CA‐CPR group; levo, levosimendan treatment group. *P < 0.05, vs. sham; ^#^ *P* < 0.05, vs. Asp. Student's *t*‐test was used to assess the difference between two groups. *P* value < 0.05 was considered statistically significant.

To determine whether levosimendan treatment exerted a renoprotective effect, we evaluated renal structural damage and function. Levosimendan treatment attenuated renal structural damage, as indicated by the decreases in tubular dilatation and atrophy, brush border loss, intraluminal cast formation, and macrophage infiltration in kidneys demonstrated by H&E staining (Fig. 2A,B). Moreover, levosimendan‐treated rats showed significant reductions in Scr and Bun levels after ROSC was achieved (Fig. 2C,D).

### Levosimendan inhibits CA‐induced renal macrophages and proinflammatory cytokines in the kidney

**Fig. 3:**
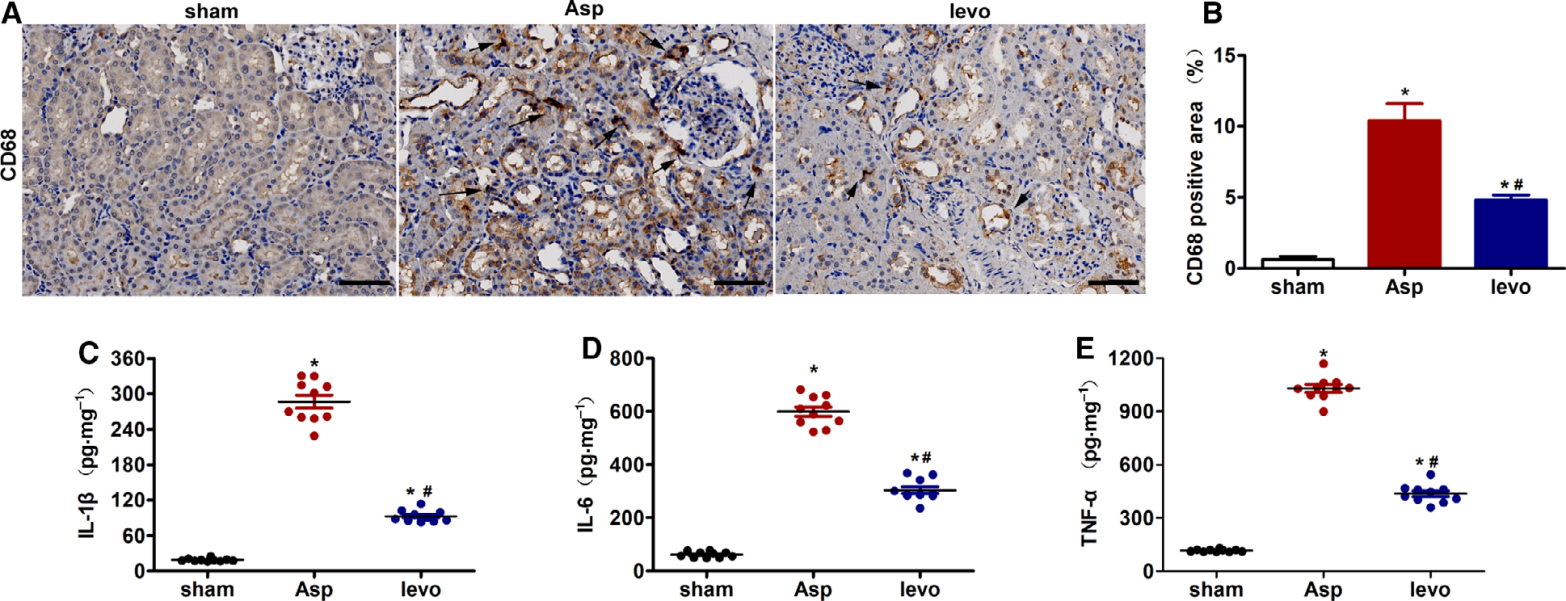
Levosimendan inhibits CA‐induced renal macrophages and proinflammatory cytokines in the kidney. (A) Representative micrographs of staining for CD68 in the kidney at 24 h after CA‐CPR (scale bar 100 μm) and (B) quantitative analysis of positive staining. Arrows indicate positive staining. (C) Proinflammatory cytokines in the kidney homogenates including IL‐1β, (D) IL‐6, and (E) TNF‐α. Data represent mean ± SEM, *n* = 6–10. Asp, CA‐CPR group; levo, levosimendan treatment group. *P < 0.05, vs. sham; ^#^ *P* < 0.05, vs. Asp. Student's *t*‐test was used to assess the difference between two groups. *P* value < 0.05 was considered statistically significant.

Macrophage involvement in CA‐induced renal injury was assessed. As shown in Fig. 3A,B, CD68‐positive macrophages were present at low levels in the sham group. Strongly stained CD68‐positive macrophages appeared in the renal interstitium and glomerulus in the Asp group. Levosimendan treatment inhibited CA‐induced CD68 expression.

To further explore the renoprotective effects of levosimendan on CA‐induced AKI, the levels of IL‐1β, IL‐6, and TNF‐α in kidney homogenates were measured by ELISA. As presented in Fig. 3C–E, in the Asp group, the expression of IL‐1β, IL‐6, and TNF‐α increased significantly (*P* < 0.01) compared with that in the sham group. However, levosimendan treatment attenuated the above proinflammatory cytokine expression compared with Asp group.

### Levosimendan reduces apoptosis in the kidneys of rats with CA‐induced AKI and H/R‐treated cells

**Fig. 4:**
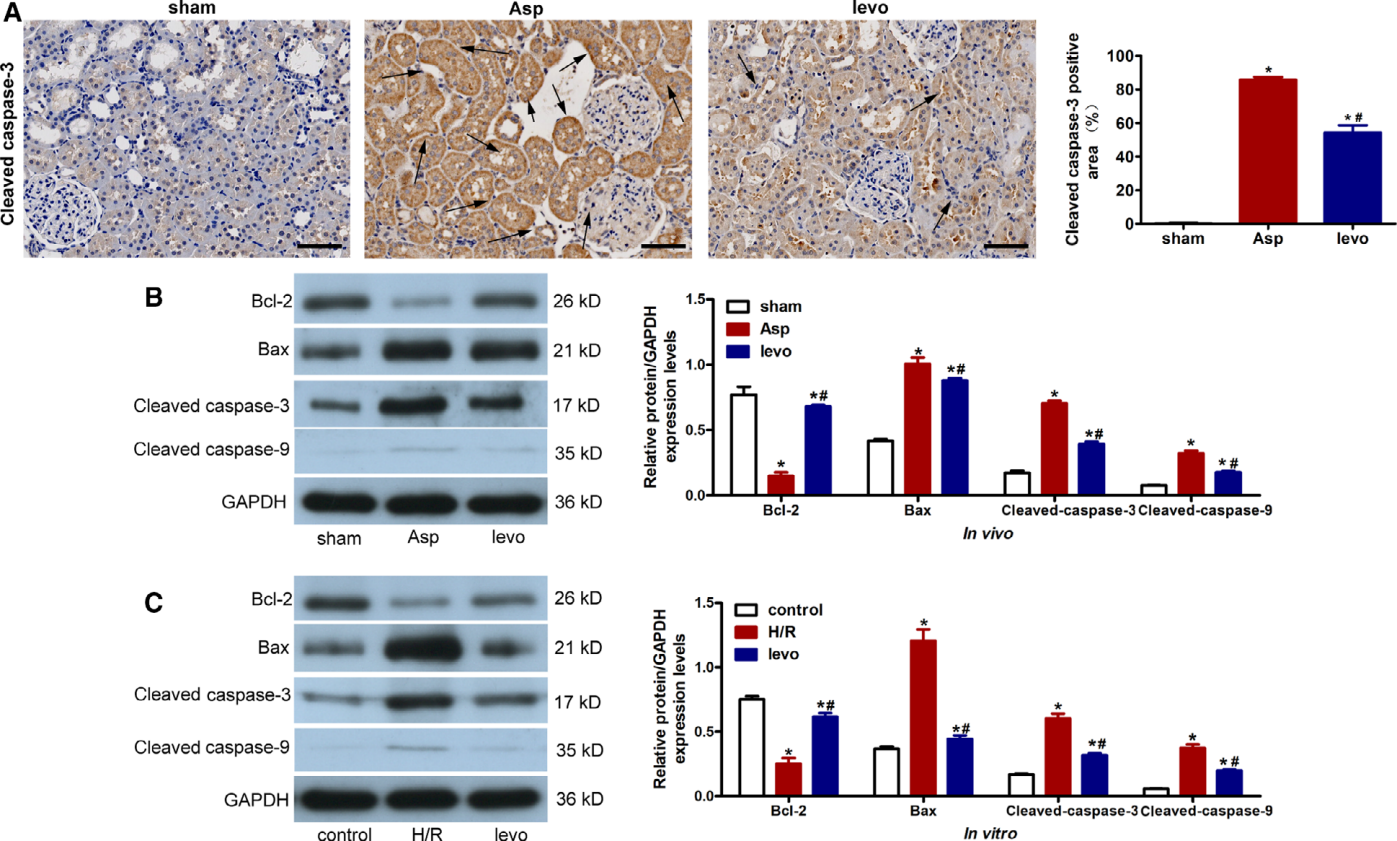
Levosimendan reduces cell apoptosis in the kidneys of rats with CA‐induced AKI and H/R‐treated cells. (A) Representative micrographs of staining for cleaved caspase‐3 in the kidney at 24 h after CA‐CPR (scale bar 100 μm) and quantitative analysis of positive staining. Arrows indicate positive staining. (B) Immunoblot analysis of renal tissue lysates from kidneys, and densitometry analysis of Bax, Bcl‐2, cleaved caspase‐3, and cleaved caspase‐9. (C) Immunoblot analysis of NRK‐52E cells in different groups and densitometry analysis of Bax, Bcl‐2, cleaved caspase‐3, and cleaved caspase‐9. Data represent mean ± SEM, *n* = 3–6. Asp, CA‐CPR group; H/R, hypoxia/reperfusion injury group; levo, levosimendan treatment group. *P < 0.05, vs. sham or control; ^#^ *P* < 0.05, vs. Asp or H/R. Student's *t*‐test was used to assess the difference between two groups. *P* value < 0.05 was considered statistically significant.

Renal cell apoptosis is associated with the pathogenesis of AKI, while Bax, Bcl‐2, cleaved caspase‐3, and cleaved caspase‐9 have been found to be involved in cell apoptosis [15, 16]. As shown in Fig. 4A by immunohistochemical staining, the expression of cleaved caspase‐3 was significantly reduced by levosimendan treatment. In Fig. 4B, it was observed that expressions of Bax, cleaved caspase‐3, and cleaved caspase‐9 were upregulated whereas Bcl‐2 expression was downregulated in CA‐induced AKI rats (*P* < 0.05, vs. sham); however, levosimendan treatment reversed these expression trends (*P* < 0.05, vs. Asp). Similarly to the *in vivo* results (Fig. 4C), the *in vitro* data presented that levosimendan pretreatment ameliorated apoptosis in H/R‐treated cells.

### Levosimendan modulates AKI via ERK activation *in vivo* and *in vitro*

**Fig. 5:**
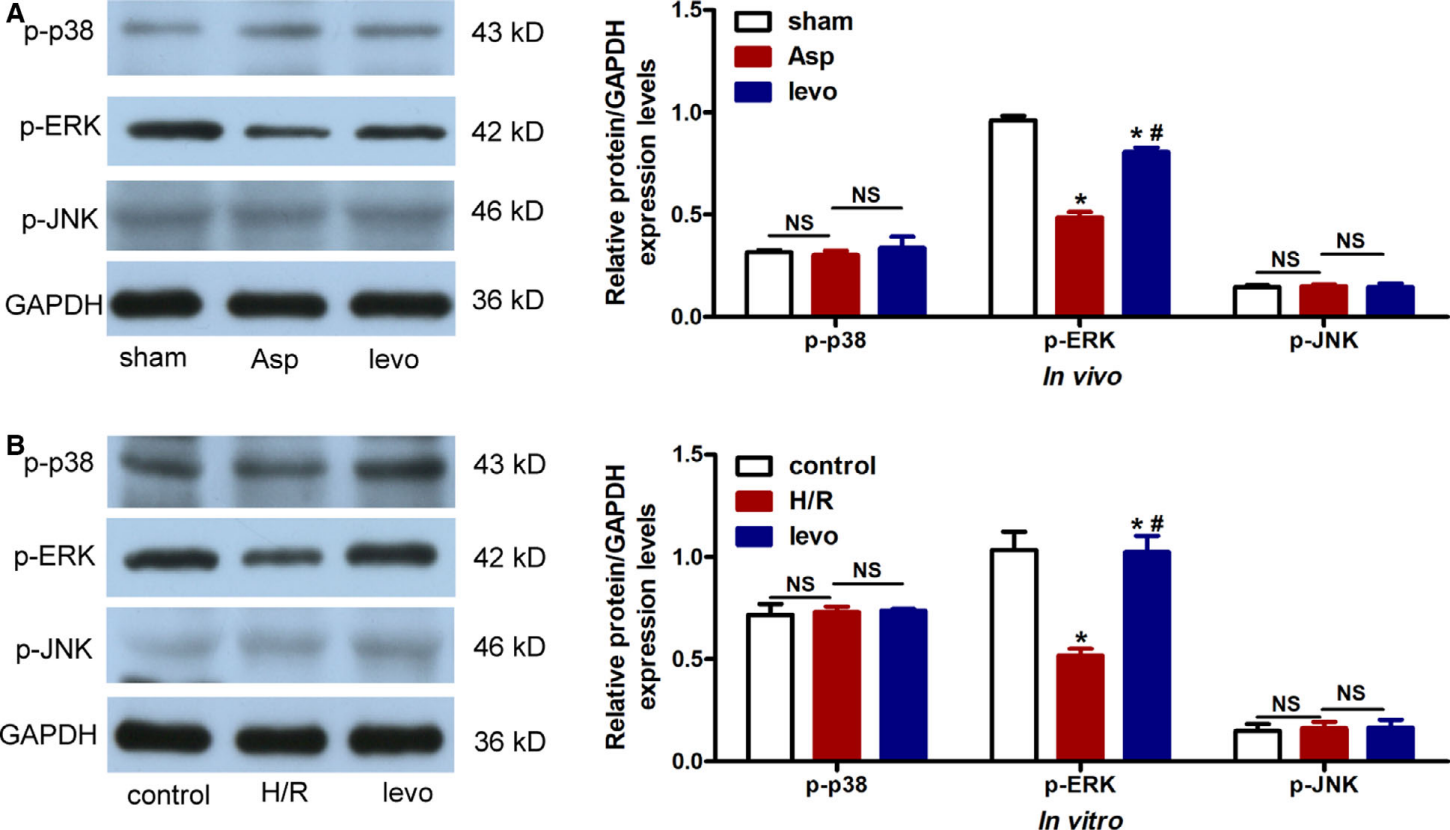
Levosimendan modulates AKI via ERK activation *in vivo* and *in vitro*. (A) Immunoblot analysis of renal tissue lysates from kidneys, and densitometry analysis of p‐p38, p‐ERK, and p‐JNK. (B) Immunoblot analysis of NRK‐52E cells in different groups, and densitometry analysis of p‐p38, p‐ERK, and p‐JNK. Data represent mean ± SEM, *n* = 3–6. Asp, CA‐CPR group; H/R, hypoxia/reperfusion injury group; levo, levosimendan treatment group. *P < 0.05, vs. sham or control; ^#^ *P* < 0.05, vs. Asp or H/R; NS, not significant. Student's *t*‐test was used to assess the difference between two groups. *P* value < 0.05 was considered statistically significant.

To identify the anti‐inflammatory and anti‐apoptotic mechanisms of levosimendan in the kidneys of rats with CA‐induced AKI and H/R‐treated cells, the mitogen‐activated protein kinase (MAPK) pathway was further explored by western blot analysis. As shown in Fig 5A, we found that p‐ERK expression was significantly suppressed after CA‐CPR (*P* < 0.05, vs. sham), which is indicative of ERK inhibition under CA‐induced AKI. Interestingly, levosimendan enhanced p‐ERK levels (*P* < 0.05, vs. Asp). As shown in Fig. 5B, the results of the *in vitro* experiments were analogous to those obtained *in vivo*, confirming the contribution of levosimendan to ERK activation.

## Discussion

Systemic ischemia–reperfusion injury (IRI) is the main pathological factor associated with the high mortality rate in patients with CA who are resuscitated, and cerebral IRI is the main cause of death and dysfunction [17]. Current studies largely focus on heart and brain injury after CPR rather than kidney injury. In recent years, many studies have reported that AKI is a common complication, with an incidence rate of approximately 12%–81% [7, 8]. A systematic review study showed that more than 50% of patients with CA developed AKI within 2 days of resuscitation [18]. Our study showed that asphyxia‐induced CA induced AKI in rats. Renal histopathology showed that CA mainly caused kidney tubular injury, one of the manifestations of IRI. AKI is an independent risk factor for death in critically ill patients. It not only increases the mortality rate of patients who are resuscitated by CPR but also uses more medical resources [19]. Therefore, more studies on acute renal injury after CPR need to be conducted.

KATP channels have been shown to be protective effectors involved in the regulation of IRI [20]. Levosimendan is a nonselective KATP channel activator. Two meta‐analyses of randomized controlled trials have shown that levosimendan not only improves AKI after cardiac surgery but also benefits patients with severe acute renal failure [21, 22]. A multicenter, randomized, double‐blind clinical trial reported that levosimendan prevented the development of AKI in patients with chronic kidney disease after mitral valve replacement [23]. Our results showed that levosimendan improved the renal function of rats after CA‐CPR and reduced renal tissue damage in rats after CA. Our results further confirm that levosimendan can protect the kidney.

Levosimendan indirectly protects the kidney by improving systemic hemodynamics (increasing cardiac output and reducing central venous pressure) and renal hemodynamics (afferent glomerular arterioles), and it also directly protects the kidney through anti‐inflammatory and anti‐apoptotic effects [24, 25]. In our study, we observed no effect of levosimendan on MAP in rats’ model of CA. Here, we focused on the direct role of levosimendan in renal tissue. A large number of studies have demonstrated that proinflammatory cytokines have prominent roles in AKI [26, 27]. Overproduction of inflammatory factors, such as IL‐1β, IL‐6, and TNF‐α, aggravated pathophysiological process of AKI [28, 29]. Therefore, anti‐inflammatory therapy could be promising for treatment of AKI. An earlier published study has confirmed that levosimendan inhibits the expression of proinflammatory factors [30]. Other studies have shown that this drug also plays an anti‐inflammatory role by inhibiting the production of reactive oxygen species [31]. The increased infiltration of inflammatory cell in the kidney induced the process of apoptosis, which in turn exacerbated renal epithelial cell loss that characterized AKI [27]. Abundant evidence has revealed that Bcl‐2 is an anti‐apoptotic protein that forms a dimer with proapoptotic protein Bax, which could promote the expression of apoptotic protein cleaved caspase‐3 and cleaved caspase‐9 [32]. A study on hypoxia–reoxygenation of human hepatocytes suggests that levosimendan produces an anti‐apoptotic effect by modulating Bax/Bcl‐2 [33]. It has also been reported in studies of lung IRI that levosimendan improves apoptosis in lung tissue [34]. The results of our study showed that levosimendan decreased the expression levels of IL‐1β, IL‐6, and TNF‐α in the kidneys of rats after CPR and improved the apoptosis of renal epithelial cells, and our results are consistent with those mentioned above.

The ERK pathway, a typical MAPK pathway, is involved in many physiological activities, including gene expression, cell cycle control, cell movement, stress‐induced inflammation, and cell apoptosis [35]. The study by Zou *et al*. [36] showed that erythropoietin activated the ERK signaling pathway to improve lung IRI. A study on hypoxia–reoxygenation of human HK‐2 cells suggested that the ERK pathway is involved in the inflammatory response and apoptosis [37]. Our animal experiments showed that the expression level of p‐ERK decreased after CPR in rats with AKI, while this level increased after treatment with levosimendan, suggesting that this drug may improve renal injury after CPR by activating the ERK pathway. These results are consistent with those of *in vitro* experiments. A recent study of cardiomyocytes reported that KATP channel opening activated the p‐ERK pathway [38]. Therefore, we hypothesize that levosimendan might improve AKI after CPR in rats by activating the ERK pathway. Previous studies have provided evidence to support the involvement of p38 and JNK in AKI models. Salama *et al*. [39] reported gentamycin‐induced AKI was associated with the activation of renal p38. In addition, JNK activation was increased in rhabdomyolysis‐induced AKI [40]. However, the activation of p38 and JNK pathway was not involved in our model. These results suggest that KATP channels may be related to ERK pathway activation. Mechanism of KATP channel interaction with ERK pathway will be deeply investigated in our further studies.

## Conclusion

In conclusion, this study reports the effects of levosimendan on AKI following CA and CPR. The present study shows that levosimendan treatment improves CA‐induced mortality and AKI. The mechanisms of its renal protection may include anti‐inflammatory and anti‐apoptotic actions by a mechanism that involves ERK signaling activation.

## Conflict of interest

The authors declare no conflict of interest.

## Author contributions

WY, CZ, XL, and HG conceived and designed the experiments. LT, SW, and LZ performed the experiments. LT, SW, and LZ analyzed the data. LT, WY, and HG wrote the manuscript.

## Data Availability

Data are available from the corresponding author upon reasonable request. Correspondence and requests for materials should be addressed to WY (yangweiqiang@renji.com) or HG (dragon_ddr@126.com).
